# Evolved polygenic herbicide resistance in *Lolium rigidum* by low-dose herbicide selection within standing genetic variation

**DOI:** 10.1111/j.1752-4571.2012.00282.x

**Published:** 2012-07-12

**Authors:** Roberto Busi, Paul Neve, Stephen Powles

**Affiliations:** 1 Australian Herbicide Resistance Initiative, School of Plant Biology, UWA Institute of Agriculture, University of Western AustraliaCrawley, WA, Australia; 2 School of Life Sciences, Warwick University, Warwick HRIWellesbourne, Warwick, UK

**Keywords:** additive genes, evolution, inheritance, Mendelian segregation, polygenic resistance

## Abstract

The interaction between environment and genetic traits under selection is the basis of evolution. In this study, we have investigated the genetic basis of herbicide resistance in a highly characterized initially herbicide-susceptible *Lolium rigidum* population recurrently selected with low (below recommended label) doses of the herbicide diclofop-methyl. We report the variability in herbicide resistance levels observed in F_1_ families and the segregation of resistance observed in F_2_ and back-cross (BC) families. The selected herbicide resistance phenotypic trait(s) appear to be under complex polygenic control. The estimation of the effective minimum number of genes (*N*_E_), depending on the herbicide dose used, reveals at least three resistance genes had been enriched. A joint scaling test indicates that an additive-dominance model best explains gene interactions in parental, F_1_, F_2_ and BC families. The Mendelian study of six F_2_ and two BC segregating families confirmed involvement of more than one resistance gene. Cross-pollinated *L. rigidum* under selection at low herbicide dose can rapidly evolve polygenic broad-spectrum herbicide resistance by quantitative accumulation of additive genes of small effect. This can be minimized by using herbicides at the recommended dose which causes high mortality acting outside the normal range of phenotypic variation for herbicide susceptibility.

## Introduction

The great advantage of herbicides is their ability to selectively remove weed plants from crop fields and for this reason herbicides are used worldwide (Oerke 2006). However, recurrent and persistent herbicide use has resulted in the evolution of resistance in many weed species (Heap 2012). The speed of resistance evolution is influenced by specifics of the herbicide selection (use history, dose applied, associated agronomic practices), the biology of the plant species under selection (population growth rate, genetic diversity, reproductive mode, etc.) and population genetic factors (Jasieniuk et al. 1996). Of the range of gene traits that can endow herbicide resistance often there is target-site resistance resulting from a point mutation in a gene encoding for a specific herbicide target enzyme (Powles and Yu 2010). Target-site resistance is thus usually single gene inherited (Darmency 1994). Conversely, resistance can be nontarget-site based. For example, cytochrome P450 mono-oxygenases or glutathione *S*-transferases can metabolize certain herbicides and evolved resistance can be due to enhanced rates of herbicide metabolism endowed by these enzymes (Yuan et al. 2007). In evolved herbicide-resistant populations of the important cross-pollinated grass weeds *Alopecurus myosuroides* (Huds.) and *Lolium rigidum* (Gaud.), enhanced rates of herbicide metabolism have been shown (Preston et al. 1996; Reade et al. 2004). In these cases of nontarget-site herbicide resistance mediated by herbicide metabolism, individual genes can endow resistance to specific herbicides (Preston 2003) and/or there can be more complex genetic linkages (Busi et al. 2011b). Diverse patterns of herbicide resistance can be evident at both the individual and population level (Petit et al. 2010a,b).

Here, we are concerned with herbicide resistance evolution from selection at low herbicide doses. Our studies conducted with *L*. *rigidum* have shown that recurrent low herbicide dose selection, within the range of quantitative variation for herbicide response evident within a small population, can rapidly lead to herbicide resistance evolution (Neve and Powles 2005; Busi and Powles 2009, 2011; Manalil et al. 2011). In small populations, a few generations of recurrent selection can lead to significant phenotypic shifts depending on the extent of intra-population genetic variation and heritability of traits. This has been shown in plants and insects and interpreted as incremental stacking of several genes of minor effect (Ffrench-Constant et al. 2004). In addition to selection, founder effects and genetic drift may influence the dynamics of resistance evolution (Falconer 1981). Here, we investigated the genetic basis of herbicide resistance as selected by low herbicide doses in cross-pollinated, genetically diverse *L. rigidum*. We report the herbicide resistance observed in the both herbicide-resistant R and initially susceptible S parental population, the variability of F_1_ families, the polygenic segregation found in F_2_ and back-cross (BC) families and discuss the management of low-dose-selected polygenic herbicide resistance.

## Materials and methods

### Plant material preparation

#### Parental lines

The well-characterized herbicide-susceptible *L. rigidum* parental population VLR1 (hereinafter referred to as S) was exposed to three cycles of recurrent selection with low (below the recommended label dose) doses of the ACCase-inhibiting herbicide diclofop-methyl (selecting agent) as described in (Neve and Powles 2005). The three-time selected line VLR1 (0.1 0.5 2.0) which exhibited the highest level of phenotypic resistance was chosen for this genetic study (hereinafter referred to as R). The coefficient 0.1, 0.5 and 2.0 represents the proportion of the recommended label dose of diclofop-methyl (375 g ha^−1^) applied to plants at the first, second and third cycles of recurrent selection, respectively. Plant survival at 0.1, 0.5 and 2.0 dose was 36%, 33% and 44%, respectively.

#### Generation of F_1_ families

R plants as described above were treated with a single herbicide dose (188 g diclofop-methyl ha^–1^) to confirm resistance, and six randomly chosen resistant R plants were each pair-crossed to one plant of the susceptible original unselected S parental to produce a total of 6 F_1_ pair crosses. Seed progeny was collected from both parental plants generating a total of 12 F_1_ families comprising six F_1_ maternal R (numbered from 1 to 6 and hereinafter referred to as ♀ R F_1_) and six reciprocal F_1_ maternal S (same numeration and hereinafter referred to as ♂ R F_1_).

#### F_1_ plant cloning to assess resistance segregation of F_1_ families

Twenty plants from each of the six ♀ R F_1_ families were grown and each individual divided into three clones for a total of 360 clones. Each series of 120 clones (20 clones by 6 F_1_ families) was treated at a different diclofop-methyl dose: 188 (low dose L), 375 (medium dose M, recommended label dose) or 1500 (high dose H) g diclofop-methyl ha^−1^. Each clone was assessed for survival 28 days after herbicide treatment. F_1_ families 5 and 6 were chosen to generate F_2_ families because, based on survival of cloned plants, there were at least two surviving plants at each L, M and H treatment.

#### Generation of F_2_ families from F_1_ cloned resistant phenotypes

Three types of F_2_ families (L, M or H) were generated by pair-crossing two cloned plants within the same F_1_ family that survived the specific L, M or H diclofop-methyl dose. Type L F_2_ families were generated by pair-crossing cloned plants from F_1_ families that were only able to survive 188 g diclofop-methyl ha^−1^ (cloned counterparts were killed at 375 and 1500 g ha^−1^). Type M F_2_ families were generated by pair-crossing cloned plants surviving 188 and 375 g diclofop-methyl ha^−1^ but killed at the highest dose (1500 g ha^−1^). Type H F_2_ families were generated by pair-crossing cloned plants that were able to survive the high dose of 1500 g diclofop-methyl ha^−1^.

#### Generation of back-cross families

Plants from ♀ R F_1_ families were treated with 188 g diclofop-methyl ha^−1^ at the two-leaf stage to eliminate susceptible individuals. The mean plant survival ratio in F_1_ families was 75%, significantly different from survival in the S (χ^2^ = 59.1; *P* < 0.01), but not significantly different to survival observed in the R plants (χ^2^ = 0.68; *P* = 0.40). This suggested the phenotypic herbicide resistance trait(s) were endowed by dominant gene(s). As suggested by Tabashnik (2001), F_1_ survivors of families 5 and 6 were pair back-crossed to plants of the original parental S population. Seeds were collected from both plants (♀, ♂ S).

### Data analysis

#### Effective number of resistance genes and scaling test analysis

The effective (minimum) number of genes (*N*_E_) for resistance to diclofop-methyl in parental, F_1_ and F_2_ families was estimated as suggested by Lande (1981) as:


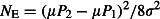
(1)

μ_P2_ – μ_P1_ represent the difference in the mean phenotypic value (aboveground plant biomass) of parental populations (R and S) and *σ*^*2*^ is the difference in variance for the same phenotypic trait between F_2_ and F_1_ families.

#### Scaling test

Additive and dominance effects of the gene(s) endowing diclofop-methyl resistance were assessed by a scaling test as described in Mather and Jinks (1982). Observed mean phenotypic values (aboveground biomass) following herbicide treatment of parental R and S, F_1_, F_2_ and BC families were tested for conformity to an additive-dominance model. The quantities *A* and *C* and their variances were calculated as described by Mather's equations (Mather and Jinks 1982) and test the adequacy of an additive-dominance model to the observed data in this study. If the model is adequate, *A* and *C* are equal to zero, and a Student's *t*-test was used to assess values for *A* and *C* were significantly different from zero. Pooled aboveground dry biomass data of F_1_ F_2_ and BC were used to calculate the parameters:


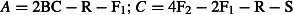
(2)

#### Maternal effects and herbicide dose–response of parental, F_1_, F_2_ and BC genetic families

The resistant parental R, the unselected susceptible parent S and four F_1_ families (no. 5 ♀, ♂ R1 F_1_, and no. 6 ♀, ♂ R2 F_1_), six F_2_ families (no. 5 and no. 6 for each family L, M and H) and two ♀ BC families (no. 5 and 6) were tested in final diclofop-methyl dose–response studies conducted at different times but under identical glasshouse conditions. Seeds were germinated on 0.6% (w/v) agar. Seedlings were transplanted and grown in 18-cm diameter pots containing a potting mix (50% peatmoss, 25% river sand and 25% pine bark) and maintained in glasshouse conditions. Plants at the two-leaf stage were treated with 0, 46, 188, 375 or 1500 g diclofop-methyl ha^−1^ (recommended label dose 375 g ha^−1^). In each experiment, there were three replicates per treatment with a minimum of 37 plants treated at each herbicide dose depending on seed availability and germination. Plant survival and aboveground dry biomass were evaluated 21 days after herbicide treatment.

As described in Busi et al. (2011b), maternal effects of diclofop-methyl resistance genes were assessed by nonlinear regression analysis. Data from the two experiments were pooled and plant survival data were expressed as percentages. Data sets were fitted to a three-parameter log-logistic model:


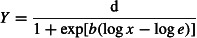
(3)

where the parameter *d* is the upper limit, *b* is the slope of the curve, *x* is the herbicide dose, and *e* is the dose producing a 50% reduction in response. Regression assumptions were held under square root data transformation (i.e. Box-Cox transformation lambda λ = 0.5) (Onofri et al. 2010). Statistical differences in plant survival between the parental and F_1_ families over a range of the herbicide diclofop-methyl doses were assessed by a lack-of-fit *F*-test applied to data sets fitted with the above nonlinear logistic model.

#### Resistance segregation in F_2_ and BC families

The segregation of genetic traits endowing diclofop-methyl resistance was assessed along a gradient of doses: 188 (low), 375 (medium) or 1500 (high) g diclofop-methyl ha^−1^. Diclofop-methyl resistance was assumed to be endowed by at least one incompletely dominant additive resistance allele. As described by Tabashnik (2001) and Preston (2003), the segregation analysis in F_2_ and BC families was based on survival as resistant (alive) or susceptible (dead) plants compared with the expected survival/mortality H_o_ ratios (Table S1). The expected survival ratio in F_2_ and BC families was corrected by weighting according to the observed performances of R and S parents and F_1_ families (Table S1). Thus, the expected F_2_ or BC survival values as number of plants are calculated with the total number of plants herbicide-treated multiplied by the theoretical one, two or three segregation ratios, respectively, (e.g. for one-gene model that ratio is 0.25R:0.5F1:0.25S) multiplied by the observed survival (%) in R, F1 and S at that specific dose (see example in Table S1). As described by Busi et al. (2011b), for each segregating F_2_ and BC family, a goodness of fit chi-square (χ^2^) test was used to compare the observed plant survival with the expected calculated values according to one-, two- or three-resistance gene segregation models (Tables 3; Table S1). *P*-values were obtained indicating the probability of type II error in rejecting the null hypothesis (H_o_ = the F_2_ family segregates as one resistance-endowing gene in a 0.25R:0.5 F_1_:0.25S ratio or two resistance genes in a 0.3125R:0.625 F_1_:0.0625S ratio or three genes in a 0.375R:0.609375 F_1_:0.015625S ratio; BC segregates as 0.5 F_1_:0.5S (1 gene), 0.75 F_1_:0.25S (2 genes) or 0.875 F_1_:0.125S (3 genes) (Table S1). The significance level was α = 0.05 (two-sided). Statistical differences between survival proportion pairs or multiple comparisons (heterogeneity test) were also assessed by χ^2^ tests performed by using the statistical software R with the command prop.test.

**Table 1:** Statistical differences between survival proportion was also assessed by chi-square tests performed by using the statistical software R with the command prop.test. A pooled chi-square value was calculated considering the sum of all the survivors in F_1_ families and a heterogeneity chi-square test was performed to compare the segregation frequencies obtained in each family (Sokal and Rohlf 1969)

F_1_ Family	Diclofop-methyl treatment (g ha^−1^)	Plants treated	Survivors observed	Expected ratio	Survivors expected	χ^2^	*P*
1	188	20	17	0.77	15.3	0.78	0.38
2	188	20	14	0.77	15.3	0.50	0.48
3	188	20	17	0.77	15.3	0.78	0.38
4	188	20	16	0.77	15.3	0.12	0.72
5	188	20	16	0.77	15.3	0.12	0.72
6	188	20	12	0.77	15.3	3.11	0.08
Total	188	120	92	0.77	92	0.00	1.00
Heterogeneity						5.40	0.37
1	375	20	14	0.6	12	0.83	0.36
2	375	20	13	0.6	12	0.21	0.65
3	375	20	15	0.6	12	1.88	0.17
4	375	20	7	0.6	12	5.21	0.02
5	375	20	13	0.6	12	0.21	0.65
6	375	20	10	0.6	12	0.83	0.36
Total	375	120	72	0.6	72	0.00	1.00
Heterogeneity						9.17	0.10
1	1500	20	11	0.33	6.6	4.38	0.04
2	1500	20	13	0.33	6.6	9.26	0.00
3	1500	20	2	0.33	6.6	4.79	0.03
4	1500	20	0	0.33	6.6	9.85	0.00
5	1500	20	9	0.33	6.6	1.30	0.25
6	1500	20	5	0.33	6.6	0.58	0.45
Total	1500	120	40	0.33	40	0.00	1.00
Heterogeneity						30.2	<0.001

**Table 2:** Herbicide resistance segregation observed in lines L, M and H of F_2_ no. 5 treated at three different diclofop-methyl doses and chi-square analysis for expected plant survival by assuming involvement of a different number of resistance genes

F_2_ family	Diclofop-methyl dose (g ha^−1^)	Plants treated	Survivors (observed)	%	Genes (*n*)	Survivors (expected)*	Segregation ratio	χ^2^	*P*
R	188	123	116	94					
R	375	150	119	79					
R	1500	159	113	71					
S	188	143	15	10					
S	375	163	10	6					
S	1500	153	2	1					
F_1_ 5	188	77	46	60					
F_1_ 5	375	84	43	51					
F_1_ 5	1500	80	39	49					
F_2_ 5L	188	37	18	49	1	20.7	1R:2 F_1_:1S	0.83	0.36
F_2_ 5 L	375	41	17	41	1	19.3	1R:2 F_1_:1S	0.50	0.48
F_2_ 5 L	1500	37	10	27	1	15.7	1R:2 F_1_:1S	3.61	0.06
F_2_ 5 M	188	67	38	57	1	37.6	1R:2 F_1_:1S	0.01	0.92
F_2_ 5 M	375	85	40	47	1	39.9	1R:2 F_1_:1S	0.00	0.99
F_2_ 5 M	1500	96	33	34	1	40.8	1R:2 F_1_:1S	2.57	0.11
F_2_ 5 H	188	81	65	80	2	63.4	5R:10 F1:1S	0.19	0.67
F_2_ 5 H	375	84	50	60	2	55.4	5R:10 F1:1S	1.55	0.21
F_2_ 5 H	1500	100	65	65	2	59.7	5R:10 F1:1S	1.15	0.28
F_2_ 5 H	188	81	65	80	3	58.3	24R:39 F1:1S	2.77	0.10
F_2_ 5 H	375	84	50	60	3	51.3	24R:39 F1:1S	0.08	0.78
F_2_ 5 H	1500	100	65	65	3	56.4	24R:39 F1:1S	3.02	0.08

* Survivors expected in F_2_ is the calculated number of plants treated multiplied by the theoretical one, two or three segregation ratios, respectively, (e.g. for one-gene model that ratio is 0.25R:0.5F1:0.25S) multiplied by the observed survival (%) in R, F1 and S at that specific dose.

**Table 3:** Herbicide resistance segregation observed in lines L, M and H of F_2_ no. 6 treated at three different diclofop-methyl doses and chi-square analysis for expected plant survival by assuming involvement of a different number of resistance genes

F_2_ family	Diclofop-methyl dose (g ha^−1^)	Plants treated	Survivors (observed)	%	Genes (*n*)	Survivors (expected)*	Segregation ratio	χ^2^	*P*
R	188	123	116	94					
R	375	150	119	79					
R	1500	159	113	71					
S	188	143	15	10					
S	375	163	10	6					
S	1500	153	2	1					
F_1_ 6	188	175	128	73					
F_1_ 6	375	180	118	66					
F_1_ 6	1500	205	94	46					
F_2_ 6 L	188	53	21	40	1	33.3	1R:2 F_1_:1S	12.15	0.00
F_2_ 6 L	375	51	20	39	1	27.6	1R:2 F_1_:1S	4.58	0.03
F_2_ 6 L	1500	48	5	10	1	19.7	1R:2 F_1_:1S	18.58	0.00
F_2_ 6 M	188	45	28	62	1	28.2	1R:2 F_1_:1S	0.01	0.94
F_2_ 6 M	375	44	18	41	1	23.8	1R:2 F_1_:1S	3.10	0.08
F_2_ 6 M	1500	47	15	32	1	19.3	1R:2 F_1_:1S	1.61	0.20
F_2_ 6 H	188	49	34	69	2	37.2	5R:10 F1:1S	0.90	0.34
F_2_ 6 H	375	52	39	75	2	34.4	5R:10 F1:1S	1.82	0.18
F_2_ 6 H	1500	50	40	80	2	25.5	5R:10 F1:1S	5.82	0.02
F_2_ 6 H	188	49	34	69	3	39.2	24R:39 F1:1S	0.07	0.79
F_2_ 6 H	375	52	39	75	3	36.3	24R:39 F1:1S	0.67	0.41
F_2_ 6 H	1500	50	40	80	3	27.3	24R:39 F1:1S	3.61	0.06

* Survivors expected in F_2_ is the calculated number of plants treated multiplied by the theoretical one, two or three segregation ratios, respectively, (e.g. for one-gene model that ratio is 0.25R:0.5F1:0.25S) multiplied by the observed survival (%) in R, F1 and S at that specific dose.

#### Herbicide cross-resistance study

In a separate study, a sub-sample of 100 seedlings of each parental R and S, F_1_ and F_2_ families was also treated with a discriminating (label) dose of the ACCase-inhibiting herbicides sethoxydim (186 g ha^−1^) or diclofop-methyl (375 g ha^−1^) or the very dissimilar ALS-inhibiting herbicides, sulfometuron (30 g ha^−1^) or chlorsulfuron (30 g ha^−1^). Survivors of chlorsulfuron and diclofop-methyl were treated with 2 kg malathion ha^−1^ followed eight hours later by another treatment with the same herbicide at the same dose. In *L. rigidum,* malathion can overcome chlorsulfuron resistance by inhibiting cytochrome P450 enzymatic activity (Christopher et al. 1994; Preston et al. 1996). Thus, malathion was used to test for a common genetic basis for cross-resistance between the very dissimilar herbicides diclofop-methyl and chlorsulfuron in R, F_1_, F_2_ and S plants. We tested whether common P450 enzymes (i.e. genes) for resistance to both herbicides would be equally inhibited by the P450 inhibitor malathion. Sethoxydim and sulfometuron were used to identify the presence of resistant individuals carrying mutations in the *ACCase* or *ALS* gene, respectively (Busi et al. 2011b). Metabolism-based resistance has never been reported for both sethoxydim and sulfometuron, and thus, survival to these herbicides is likely due to occurrence of mutant alleles in *ACCase* or *ALS* genes, respectively (Christopher et al. 1991; Tardif et al. 1993).

## Results

### Minimum gene number and additive-dominance model

The estimation of the minimum number of resistance genes (*N*_E_) indicated that, at the population level, at least three resistance genes were present in the original parental herbicide-susceptible (S) *L. rigidum* population. Most probably, all three genes were not all initially stacked within S individuals; however, over three cycles of recurrent low-dose diclofop-methyl selection, these three resistance genes became accumulated within surviving individuals through cross-pollination. Once the three resistance genes were present within individuals, then they collectively endowed relatively high-level diclofop-methyl resistance. Pooled data obtained from parental families (R and S) and respective F_1_, F_2_ and BC crosses at the three diclofop-methyl doses tested were assessed by a joint scaling test. Student's *t*-test revealed no significant deviation from zero of quantities *A* and *C* calculated in the scaling test (Table 4). Thus, these results indicate that gene interactions are well explained by an additive-dominance model at each diclofop-methyl dose used in the Mendelian segregation analysis.

**Table 4:** Scaling test to assess additive-dominance relationship between means (plant aboveground biomass) of different lines treated at three different diclofop-methyl doses. Data pooled for parental R1, R2 and respective F_1_, F_2_ and BC lines. Student's *t*-test was used to evaluate significant deviations from zero of *A* and *C* quantities. Minimum number of resistance genes (*N*_E_) has been estimated for each herbicide dose

Family	Diclofop-methyl treatment (g ha^−1^)	Mean plant biomass (g)	Variance	Sample size	*t*	*P*	*N*_E_
R	188	1.12	0.08				
S	188	0.05	0.00				
F_1_	188	1.04	0.27				
F_2_	188	0.87	0.15				
BC	188	0.72	0.11				
*A*	188	0.35	0.73	21	0.41	0.69	
*C*	188	0.22	3.61	40	0.12	0.91	1.2
R	375	1.20	0.06				
S	375	0.03	0.00				
F_1_	375	0.90	0.19				
F_2_	375	0.65	0.14				
BC	375	0.41	0.07				
*A*	375	−0.11	0.48	21	0.16	0.87	
*C*	375	−0.43	3.04	41	0.24	0.81	3.3
R	1500	0.80	0.17				
S	1500	0.01	0.00				
F_1_	1500	0.92	0.43				
F_2_	1500	0.52	0.14				
BC	1500	0.38	0.10				
*A*	1500	−0.17	0.84	19	0.19	0.85	
*C*	1500	−0.56	4.17	40	0.28	0.78	0.3

BC, back-cross.

### Herbicide dose–response and genetic analysis of F_1_ families

**Figure 1:**
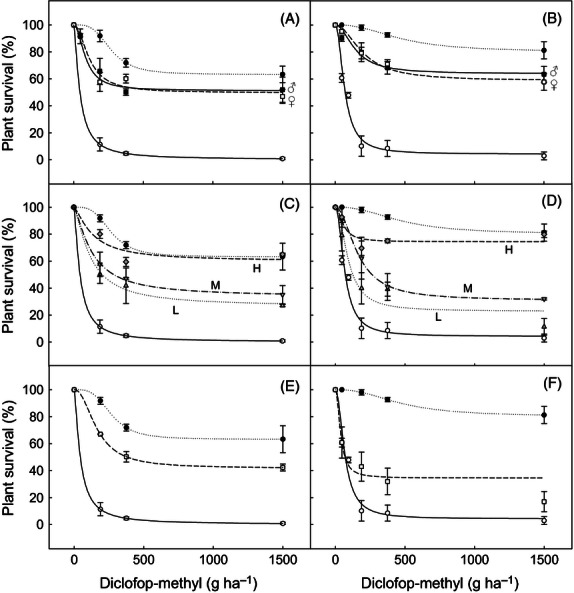
Survival response to a range of doses of diclofop-methyl for resistant parental lines (R) (solid circles and dotted line), susceptible original parental line (S) (open circles and solid line), F_1_ no. 5 (♂ solid squares and solid line), (♀ open squares and short-dashed line) (A); F_1_ no. 6 (♂ solid squares and solid line), (♀ open squares and short-dashed line) (B); F_2_ family no.5 (C); F_2_ family no. 6 (D); type L (grey triangle up and dotted lines), type M (grey triangle down and dash-dotted lines) and type H (grey diamonds and short-dashed line). back-cross (BC) no. 5 (E) and BC no. 6 (F) (open squares and short-dashed line). Symbols are mean of observed plant survival ± SE (*n* = 3). Lines are estimated plant survival following nonlinear regression analysis.

As expected, the herbicide dose–response study confirmed the R parent as diclofop-methyl resistant and the original parental S population as susceptible (Fig. 1). The comparison between dose–response curves of paternal versus maternal F_1_ families confirmed pollen-transmitted, nuclear inheritance of herbicide resistance traits without maternal effects (*P* > 0.48) (Fig. 1A,B). Overall, the diclofop-methyl resistance level observed in F_1_ families was lower than the respective R parent (*P* < 0.01).

**Figure 2:**
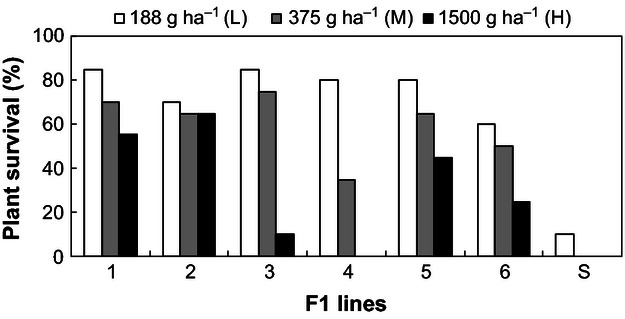
Variability in survival of 20 cloned F_1_ plants treated with 188 (Low; white bars), 375 (Medium; grey bars) and 1500 (High; black bars) g diclofop-methyl ha^−1^. Surviving cloned plants were employed to generate type L, M, H F_2_ lines in different pair crosses.

F_1_ cloned plants were confirmed to be diclofop-methyl resistant and cloned S confirmed as susceptible (Fig. 2). Overall, F_1_ plants exhibited variability in response to herbicide treatment. Using cloned plants, it was possible to show the variability evident within and between F_1_ families (Fig. 2). At the highest herbicide dose tested (H), significant heterogeneity between F_1_ families was evident, with three different response patterns. Higher survival than expected was observed in F_1_ family no.1 and 2, versus lower than expected survival in family no. 3 and 4 and with no significant deviations from the pooled mean survival in families no. 5 and 6 (Table 1). This was envisaged as we expected genetic variability between parental R plants used to generate F_1_ families. F_1_ families no. 5 and 6 were chosen to generate F_2_ plants because there was a significant segregation (*P* = 0.005) between plant response at treatments with L versus H dose (188 vs 1500 g diclofop-methyl ha^−1^). The other F_1_ families were not studied further because either this difference in herbicide response was not significant (*P* = 0.79) or there were no survivors at the H dose (Fig. 2).

### Herbicide dose–response and genetic analysis with F_2_ and BC families

#### F_2_ families

Genetic analysis indicated two different phenotypic responses in families L, M and H of F_2_ no. 5 (Fig. 1C). Lack-of-fit F-test revealed no significant difference between nonlinear models fitted to L and M F_2_ data (*F* = 1.17; *P* = 0.35). Similarly, the chi-square test revealed that a one-gene model best fitted the data obtained with families L and M. For these two families, any other genetic model involving a greater number of genes did not fit the data (Table 2). The family H exhibited a significant greater level of phenotypic resistance than families L and M (Fig. 1C) (*F* = 12.5; *P* = 0.002). Consistently, the one-gene model poorly fitted the segregation data in the F_2_ no. 5 family H (*P* < 0.02), whereas two-gene and three-gene models were found to be appropriate with no significant statistical deviations (*P* > 0.13) (Table 2). F_2_ no. 6 families exhibited three different levels of diclofop-methyl resistance (Fig. 1D). Family L had the lowest resistance level, although the lack-of-fit test did not reveal a significant difference between curves fitted to families L and M (*F* = 1.49; *P* = 0.24). The observed survival in family L was significantly lower than expected by the one-gene model at doses 375 and 1500 g diclofop-methyl ha^−1^ (Table 3). Family M showed greater level diclofop-methyl resistance and a one-gene model best fitted the observed plant survival at the three different diclofop-methyl doses (Table 3). Two- and three-gene models did not fit the data obtained with the M family (*P* < 0.01). Family H exhibited the highest resistance level (*F* = 7.47; *P* = 0.001) (Fig. 2D). Either a two- or a three-gene model had the best fit with the data (*P* > 0.08) (Table 3).

#### Back-cross families

As expected, diclofop-methyl resistance was evident in two BC families tested (Fig. 1F,G). The overall resistance level observed in BC no. 5 did not significantly decrease compared with the respective F_1_ no. 5 lines (*P* = 0.40), whereas BC no. 6 exhibited a significantly lower resistance level than F_1_s no. 6 (*P* < 0.001) (Fig. 1E,F). The segregation analysis suggests two genes were associated with resistance in BC family no. 5, similar to that observed in the F_2_ family H. A one-gene model was found to best fit the BC family no. 6 as in the F_2_ family M (Table 5). It is emphasized that F_1_ plants used to generate BC families survived at 188 diclofop-methyl only and survival at higher doses could not be tested.

**Table 5:** Herbicide resistance segregation observed in BC families no. 5 and 6 at three different diclofop-methyl doses and chi-square analysis for expected plant survival by assuming different genetic models

BC family	Diclofop-methyl dose (g ha^−1^)	Plants treated	Survivors (observed)	Genes (*n*)	Survivors (expected)*	Segregation ratio	χ^2^	*P*
BC 5	188	91	61	2	65.5	3 F_1_:1S	1.09	0.30
BC 5	375	104	52	2	50.6	3 F_1_:1S	0.08	0.78
BC 5	1500	91	39	2	45.2	3 F_1_:1S	1.68	0.19
BC 6	188	115	50	1	48.1	1 F_1_:1S	0.13	0.72
BC 6	375	136	43	1	48.7	1 F_1_:1S	1.06	0.30
BC 6	1500	132	23	1	31.1	1 F_1_:1S	2.78	0.10

BC, back-cross.

* Survivors expected in BC is the calculated number of plants treated multiplied by the theoretical one, two or three segregation ratios, respectively, (e.g. for one-gene model that ratio is 0.25R:0.5F1:0.25S) multiplied by the observed survival (%) in R, F_1_ and S at that specific dose.

### Genetic basis of herbicide cross-resistance

**Figure 3:**
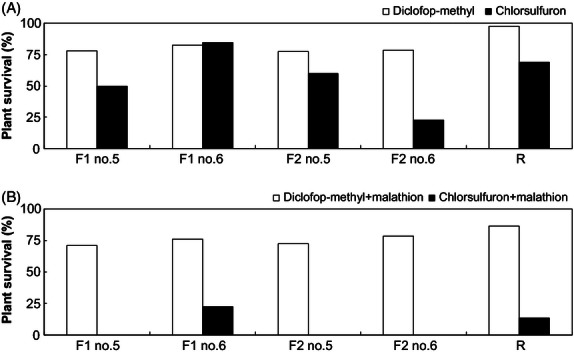
Cross-resistance between diclofop-methyl and chlorsulfuron observed in parental R and F_1_, H F_2_ families following treatments with the recommended dose of diclofop-methyl (375 g ha^−1^; white bars) or chlorsulfuron (30 g ha^−1^; black bars).

Importantly, recurrent selection at low diclofop-methyl dose resulted not only in diclofop-methyl resistance but in a level of cross-resistance to a dissimilar herbicide (chlorsulfuron) of different mode of action. Here, parental R, F_1_ and F_2_ (H type) displayed diclofop-methyl resistance and a variable degree of cross-resistance to the dissimilar ALS-inhibiting herbicide chlorsulfuron (Fig. 3A). It is emphasized that before recurrent diclofop-methyl low-dose selection, the parental S population was chlorsulfuron sensitive. Therefore, the diclofop-methyl resistance resulting from recurrent low-dose diclofop-methyl selection concomitantly resulted in chlorsulfuron resistance. A common linkage between these two otherwise very dissimilar herbicides is that both are metabolized by P450 enzymes (Busi et al. 2011b) and it has been documented in *L. rigidum* that malathion synergises chlorsulfuron because malathion can inhibit cytochrome P450 enzymes able to metabolize chlorsulfuron (Christopher et al. 1994). Therefore, if the exact same P450 enzyme(s) metabolize both diclofop-methyl and chlorsulfuron, then malathion should be able to synergize both herbicides. This was tested by first treating with either diclofop-methyl or chlorsulfuron, observing plant survival (resistant plants) then treating with diclofop-methyl or chlorsulfuron plus malathion. As expected, plants surviving chlorsulfuron alone (57% mean plant survival) were killed by a subsequent treatment with chlorsulfuron plus malathion (7% survival). However, malathion could not synergize diclofop-methyl as survivors from diclofop-methyl treatment (83% survival) had 77% survival (χ^2^ = 2.03; *P* = 0.15) by a subsequent diclofop-methyl plus malathion treatment (Fig. 3B). This indicates that the genes endowing resistance to diclofop-methyl are likely different to those endowing resistance to chlorsulfuron. Thus, low-dose selection with diclofop-methyl, enriched genes that confer diclofop-methyl resistance and other genes that confer chlorsulfuron resistance.

It is important to note that the diclofop-methyl resistance and the chlorsulfuron resistance were not ACCase or ALS target-site gene based. Sulfometuron (30 g ha^−1^) resulted in 100% mortality in R, S, F_1_ and F_2_ lines and likewise sethoxydim (186 g ha^−1^) killed all the plants treated as they do not have a resistance-endowing mutation in the *ALS* or *ACCase* gene, respectively (Neve and Powles 2005; Yu et al. 2008).

## Discussion

A highly characterized herbicide-susceptible *L. rigidum* population when subjected to directional selection for three generations with low (below-label) dose of the herbicide diclofop-methyl evolved a high level of phenotypic resistance (Fig. 1) (Neve and Powles 2005). Neve and Powles (2005) hypothesized that when herbicide selection occurs at low dose (within genetic variation at putatively many loci for herbicide response in a S population) then polygenic herbicide resistance can evolve, especially in a cross-pollinated genus like *Lolium*. By contrast, the majority of the evolved cases of herbicide resistance in *L. rigidum* and other weed species is endowed by genes of major effect, typically a mutation in the herbicide target-site (Délye 2005; Yu et al. 2007; Powles and Yu 2010). Monogenic responses are also common in other human-mediated selective systems (Roush and McKenzie 1987; Palumbi 2001). It is argued that selection of pre-existing genetic variation may result in more rapid evolution because it is immediately available in response to selection, whereas there would be no time to allow new beneficial mutation to arise (Gomulkiewicz et al. 2010). Similarly, the results of this study suggest the relative greater importance of standing genetic variation versus novel mutations when herbicide selection occurs at low dose on a relatively limited number of individuals and highly resistant phenotypes are very rare. It is emphasized that the *L. rigidum* S population used in this study was well characterized and before selection did not contain major gene(s) endowing herbicide resistance (Yu et al. 2007). Most probably, single major and minor genes can additively and/or synergistically interact to endow a level of resistance allowing plants to survive herbicide field applications. As suggested by Neve et al. (2009), it is possible that herbicide resistance evolution occurs through a ‘step-by-step’ pathway involving mainly polygenes in the early phase of selection before resistant phenotypes are evident in the field. A similar pathway was also suggested as genetic mechanism for evolution of mimicry in insects (Orr and Coyne 1992). Our study indicates that a few quantitative genes of small effect present in a small unselected herbicide-susceptible *L. rigidum* population were enriched by directional low-dose herbicide selection and these genes accumulated through cross-pollination among survivors to ultimately endow a substantial level of resistance. By contrast, herbicide resistance evolution because of gene stacking seems unlikely to occur in a self-fertilized species as shown in *Arabidopsis thaliana* L. following seven cycles of recurrent selection at low doses of glyphosate (Brotherton et al. 2007). Genetic drift can also cause significant shift in small populations (Falconer 1981). However, in the *L. rigidum* population analysed here, the persistent herbicide selection to produce resistant progeny likely prevented random loss of resistance alleles and resulted in high-level phenotypic resistance to diclofop-methyl (Neve and Powles 2005). Notably, recurrent selection with low diclofop-methyl dose resulted not only in diclofop-methyl resistance but cross-resistance to the ALS herbicide chlorsulfuron, a chemically unrelated and dissimilar herbicide mode of action. This diclofop-methyl and chlorsulfuron cross-resistance was not because of target-site mutations in either the *ACCase* or *ALS* gene as also established by previous extensive molecular analysis and biochemical work (Neve and Powles 2005; Yu et al. 2007). *Lolium rigidum* can detoxify diclofop-methyl and chlorsulfuron through P450-mediated metabolism (Christopher et al. 1991; Preston et al. 1996; Preston and Powles 1998), and we have evidence of enhanced rates of diclofop-methyl metabolism in these low-dose-selected resistant plants (Yu et al., unpublished manuscript). However, other resistance-endowing mechanism(s) cannot be ruled out. For example, gene amplification was documented as a major force for the evolution of resistance in bacteria, insects and plants (Sandegren and Andersson 2009; Gaines et al. 2010; Bass and Field 2011).

Our study here adds to only a small number of studies that have reported polygenic control of evolved herbicide resistance in weeds under herbicide selection (Letouze and Gasquez 2001; Preston 2003; Petit et al. 2010a,b; Busi et al. 2011b). In this study, the *L. rigidum* R plants exhibited high-level diclofop-methyl resistance, and cloned individuals from F_1_ families treated at different doses of diclofop-methyl showed genetic complexity by segregation of different resistant phenotypes (Fig. 2). We tested whether the three different levels of resistance to diclofop-methyl in cloned F_1_ plants would have resulted in gene segregation in F_2_ families with different levels of inherited resistance. Within each F_1_ family, we confirmed that the variability in response to diclofop-methyl dose was heritable with a more resistant F_2_ progeny generated from F_1_ phenotypes able to survive an incrementally higher diclofop-methyl dose (Fig. 1). The observed resistance levels obtained in segregating F_2_ and BC families are consistent with the hypothesis of polygenic resistance selected by enrichment (accumulation) of quantitative genes (Neve and Powles 2005; Busi and Powles 2009; Manalil et al. 2011, Manalil et al. 2012). Genetic analyses conducted in this study have clearly shown that there is involvement of more than one gene, and probably, a minimum of three effective resistance genes were enriched from the original parental S population. A population genetic analysis conducted with the polygenic modelling platform qu-gene (Podlich and Cooper 1998) also indicates polygenic resistance. A theoretical population shift was simulated by qu-gene under a range of parameters and the assumption of additive genes (see Data S1 for methodology). The results obtained by modelling simulations were compared with the observed population shift reported by Neve and Powles (2005) and similar evolutionary dynamics (observed versus simulated) were obtained under the assumption of two or three genes involved in diclofop-methyl resistance. Conversely, a greater number of resistance genes would have not resulted in similar and rapid population shifts (Figs S1–S5).

Busi et al. (2011b) reported evidence of genetic linkage between P450 genes for diclofop-methyl and chlorsulfuron resistance in a field-evolved resistant *L. rigidum* population. Here, in the S population, diclofop-methyl recurrent selection has increased the gene frequency for resistance to both ACCase- and ALS-inhibiting herbicides (diclofop-methyl and chlorsulfuron) in the R, F_1_ and F_2_ lines. Thus, we hypothesize that these are P450 genes also because diclofop-methyl and chlorsulfuron are known to be P450-metabolized in wheat (Werck-Reichhart et al. 2000). However, the different plant response observed with malathion (absence or presence of synergism between malathion and diclofop-methyl or chlorsulfuron, respectively) suggests that efficient gene(s) endowing diclofop-methyl resistance may be different to gene(s) endowing chlorsulfuron resistance. This supports the hypothesis of polygenic control of resistance in this population and presence of complex gene diversity to endow resistance across different herbicide classes (Manalil et al. 2011).

Herbicide use at low dose allows the selection of all genetic trait(s) that minimize herbicide damage and thus endow plant survival. On the contrary, use of high (recommended) herbicide doses may eliminate those weakly endowing resistance gene traits. Herbicide resistance evolution in weed species is a global challenge in modern agro-ecosystems and increasingly evolutionary concerns are raised for the sustainability of world food production in a changing climate (Neve et al. 2009; Powles and Yu 2010; Thrall et al. 2011). It appears that relatively small *L. rigidum* populations have great evolutionary potential and can quickly adapt to harsh selective environments. An immediate applied conclusion from this research is that avoiding low-dose herbicide usage could be one component of helping achieve herbicide sustainability. Herbicide use at low doses and/or under conditions that result in substantial plant survival increases the risks of enrichment of minor gene traits leading to herbicide resistance. As more information on the complex genetic basis of herbicide resistance is unravelled, it becomes evident that herbicides should be used at doses that cause very high weed mortality, and with herbicide diversity and use of nonchemical weed control strategies to minimize the likelihood of resistance genes being passed to the next generation.

## Data archiving statement

Data for this submission available as supplementary material hosted by Wiley Online.

## Supporting Information

**Figure S1.** Plant survival observed at 375 g diclofop ha^−1^ in the susceptible VLR1 parent population and the progenies 1 (VLR1 0.1), 2 (VLR1 0.1 0.5) and 3 (VLR1 0.1 0.5 2.0) subjected to recurrent selection at the reduced doses of diclofop-methyl.

**Figure S2.** Response to selection simulated with QU-GENE model under the hypothesis of completely additive model (Podlich and Cooper 1998). Three generations of recurrent selection, two additive genes, population size of 100 plants, initial gene frequency of 0.06 (population mean as plant survival in the unselected population VLR1 at 375 g diclofop-methyl ha^−1^), selection proportion 0.2, heritability 50% and three generations of recurrent selection.

**Figure S3.** Response to selection simulated with QU-GENE model under the hypothesis of completely additive model (Podlich and Cooper 1998). Three generations of recurrent selection, three additive genes, population size of 100 plants, initial gene frequency of 0.06 (population mean as plant survival in the unselected population VLR1 at 375 g diclofop-methyl ha^−1^), selection proportion 0.2, heritability 50% and three generations of recurrent selection.

**Figure S4.** Response to selection simulated with QU-GENE model under the hypothesis of completely additive model (Podlich and Cooper 1998). Three generations of recurrent selection, 10 additive genes, population size of 100 plants, initial gene frequency of 0.06 (population mean as plant survival in the unselected population VLR1 at 375 g diclofop-methyl ha^−1^), selection proportion 0.2, heritability 50% and three generations of recurrent selection.

**Figure S5.** Response to selection simulated with QU-GENE model under the hypothesis of completely additive model (Podlich and Cooper 1998). Three generations of recurrent selection, 10 additive genes, population size of 100 plants, initial gene frequency of 0.06 (population mean as plant survival in the unselected population VLR1 at 375 g diclofopmethyl ha^−1^), selection proportion 0.2, heritability 50% and three generations of recurrent selection.

**Table S1.** Genotypic segregation pattern and expected phenotypic survival in F_2_ and BC families according to one-gene (A), two-gene (B) and three-gene (C) resistance models.

**Data S1.** Metadata sets.

**Data S2.** Methodology.
