# Supplementary material for: Evolved polygenic herbicide resistance in Lolium rigidum by low-dose herbicide selection within standing genetic variation
Source: Evol Appl. 2012 Jul 12;6(2):231–42. doi: 10.1111/j.1752-4571.2012.00282.x (PMC3689349; doi:10.1111/j.1752-4571.2012.00282.x)
Supplement: Supplementary file 1 [file eva0006-0231-SD1.doc]

**Table S1** Genotypic segregation pattern and expected phenotypic survival in F2 and BC families according to one-gene (A), two-gene (B) and three-gene (C) resistance models. The capital letter “R” indicates a resistance alleles and the small capital “r” a susceptible allele. Absence of resistance allele gives does not endow herbicide resistance and the genotypic combinations are not highlighted. The genotypic combinations highlighted in light grey indicate the presence of dominant alleles (R1, R2 and R3) to endowed resistance equally as observed in F1 families. Genotypes dark grey highlighted endowed resistance equally as observed in the parental resistant line.

| A = One gene control | | |
| --- | --- | --- |
| **Alleles** | ***R1*** | ***r1*** |
| ***R1*** | *R1R1* | *R1r1* |
| ***r1*** | *r1R1* | *r1r1* |
| *F2 genotypic segregation 1R:2F1:1S (one gene)* | | |
|  |  |  |
| **Alleles** |  | ***r1*** |
| ***R1*** |  | *R1r1* |
| ***r1*** |  | *R1r1* |
| *BC genotypic segregation 1F:1S (one gene)* | | |

Worked example referred to Table 2 in the manuscript

The expected F2 or BC survival values are calculated with the number of plants herbicide-treated multiplied by the theoretical 1, 2 or 3 segregation ratios, respectively, (e.g. for 1 gene model that ratio is 0.25R:0.5F1:0.25S) multiplied by the observed survival ratio observed in R, F1 and S at that specific dose. In case of 1 gene control with segregation ratio 0.25R:0.5F1:0.25S. For each dose the theoretical survival given by the 1 gene control ratio (0.25R:0.5F1:0.25S) is corrected by weighting according to the observed performances (survival) of R and S parents and F1 components observed each specific dose and multiplied by the total number of plants herbicide-treated (37 in both cases as reported in Table 2).

**1) At 188 g diclofop-methyl ha^-1^ for F_2_ 5L**

37 x 0.94 x 0.25 + 37 x 0.6 x 0.5 + 37 x 0.1 x 0.25 = 20.7 expected number of plants compared to the 18 survived (observed)

**2) At 1500 g diclofop-methyl ha^-1^ for F_2_ 5L**

37 x 0.71 x 0.25 + 37 x 0.49 x 0.5 + 37 x 0.01 x 0.25 = 15.7 expected number of plants compared to the 10 survived (observed)

| B = Two gene control | | | | |
| --- | --- | --- | --- | --- |
| **Alleles** | ***R1R2*** | ***R1r2*** | ***r1R2*** | ***r1r2*** |
| ***R1R2*** | *R1R1 R2R2* | *R1R1 R2r2* | *R1r1 R2R2* | *R1r1 R2r2* |
| ***R1r2*** | *R1R1 R2r2* | *R1R1 r2r2* | *R1r1 R2r2* | *R1r2 r2r2* |
| ***r1R2*** | *R1r1 R2R2* | *R1r1 R2r2* | *r1r1 R2R2* | *r1r1 R2r2* |
| ***r1r2*** | *R1r1 R2r2* | *R1r1 r2r2* | *r1r1 R2r2* | *r1r1 r2r2* |
| *F2 genotypic segregation 5R:10F1:1S (two genes)* | | | | |
|  |  |  |  |  |
| **Alleles** |  |  |  | ***r1r2*** |
| ***R1R2*** |  |  |  | *R1r1 R2r2* |
| ***R1r2*** |  |  |  | *R1r2 r2r2* |
| ***r1R2*** |  |  |  | *r1r1 R2r2* |
| ***r1r2*** |  |  |  | *r1r1 r2r2* |
| *BC genotypic segregation 3F1:1S ( two-genes)* | | | | |

| C = Three gene control | | | |  |  |  |  |  |
| --- | --- | --- | --- | --- | --- | --- | --- | --- |
| **Alleles** | ***R1R2R3*** | ***R1R2r3*** | ***R1r2r3*** | ***R1r2R3*** | ***r1R2R3*** | ***r1R2r3*** | ***r1r2R3*** | ***r1r2r3*** |
| ***R1R2R3*** | *R1R1R2R2R3R3* | *R1R1R2R2R3r3* | *R1R1R2r2R3r3* | *R1R1R2r2R3R3* | *R1r1R2R2R3R3* | *R1r1R2R2R3r3* | *R1r1R2r2R3R3* | *R1r1R2r2R3r3* |
| ***R1R2r3*** | *R1R1R2R2R3r3* | *R1R1R2R2r3r3* | *R1R1R2r2r3r3* | *R1R1R2r2R3r3* | *R1r1R2R2R3r3* | *R1r1R2R2r3r3* | *R1r1R2r2R3r3* | *R1r1R2r2r3r3* |
| ***R1r2r3*** | *R1R1R2r2R3r3* | *R1R1R2r2r3r3* | *R1R1r2r2r3r3* | *R1R1r2r2R3r3* | *R1r1R2r2R3r3* | *R1r1R2r2r3r3* | *R1r1r2r2R3r3* | *R1r1r2r2r3r3* |
| ***R1r2R3*** | *R1R1R2r2R3R3* | *R1R1R2r2R3r3* | *R1R1r2r2R3r3* | *R1R1r2r2R3R3* | *R1r1R2r2R3R3* | *R1r1R2r2R3r3* | *R1r1r2r2R3R3* | *R1r1r2r2R3r3* |
| ***r1R2R3*** | *R1r1R2R2R3R3* | *R1r1R2R2R3r3* | *R1r1R2r2R3r3* | *R1r1R2r2R3R3* | *r1r1R2R2R3R3* | *r1r1R2R2R3r3* | *r1r1R2r2R3R3* | *r1r1R2r2R3r3* |
| ***r1R2r3*** | *R1r1R2R2R3r3* | *R1r1R2R2r3r3* | *R1r1R2r2r3r3* | *R1r1R2r2R3r3* | *r1r1R2R2R3r3* | *r1r1R2R2r3r3* | *r1r1R2r2R3r3* | *r1r1R2r2r3r3* |
| ***r1r2R3*** | *R1r1R2r2R3R3* | *R1r1R2r2R3r3* | *R1r1r2r2R3r3* | *R1r1r2r2R3R3* | *r1r1R2r2R3R3* | *r1r1R2r2R3r3* | *r1r1r2r2R3R3* | *r1r1r2r2R3r3* |
| ***r1r2r3*** | *R1r1R2r2R3r3* | *R1r1R2r2r3r3* | *R1r1r2r2r3r3* | *R1r1r2r2R3r3* | *r1r1R2r2R3r3* | *r1r1R2r2r3r3* | *r1r1r2r2R3r3* | *r1r1r2r2r3r3* |
| *F2 genotypic segregation****:*** 24R:39F1:1S *(three genes)* | | | | | | | | |
|  |  |  |  |  |  |  |  |  |
| **Alleles** |  |  |  |  |  |  |  | ***r1r2r3*** |
| ***R1R2R3*** |  |  |  |  |  |  |  | *R1r1R2r2R3r3* |
| ***R1R2r3*** |  |  |  |  |  |  |  | *R1r1R2r2r3r3* |
| ***R1r2r3*** |  |  |  |  |  |  |  | *R1r1r2r2r3r3* |
| ***R1r2R3*** |  |  |  |  |  |  |  | *R1r1r2r2R3r3* |
| ***r1R2R3*** |  |  |  |  |  |  |  | *r1r1R2r2R3r3* |
| ***r1R2r3*** |  |  |  |  |  |  |  | *r1r1R2r2r3r3* |
| ***r1r2R3*** |  |  |  |  |  |  |  | *r1r1r2r2R3r3* |
| ***r1r2r3*** |  |  |  |  |  |  |  | *r1r1r2r2r3r3* |
|  |  |  |  |  |  |  |  |  |
| *BC genotypic segregation 8F1:1S (three genes)* | | | | | | | | |

**Figure S1**. Plant survival observed at 375 g diclofop ha^-1^ in the susceptible VLR1 parent population and the progenies 1 (VLR1 0.1), 2 (VLR1 0.1 0.5) and 3 (VLR1 0.1 0.5 2.0) subjected to recurrent selection at the reduced doses of diclofop-methyl. This observed plant survival needs to be compared with the accumulated population mean obtained by simulation modeling.

**
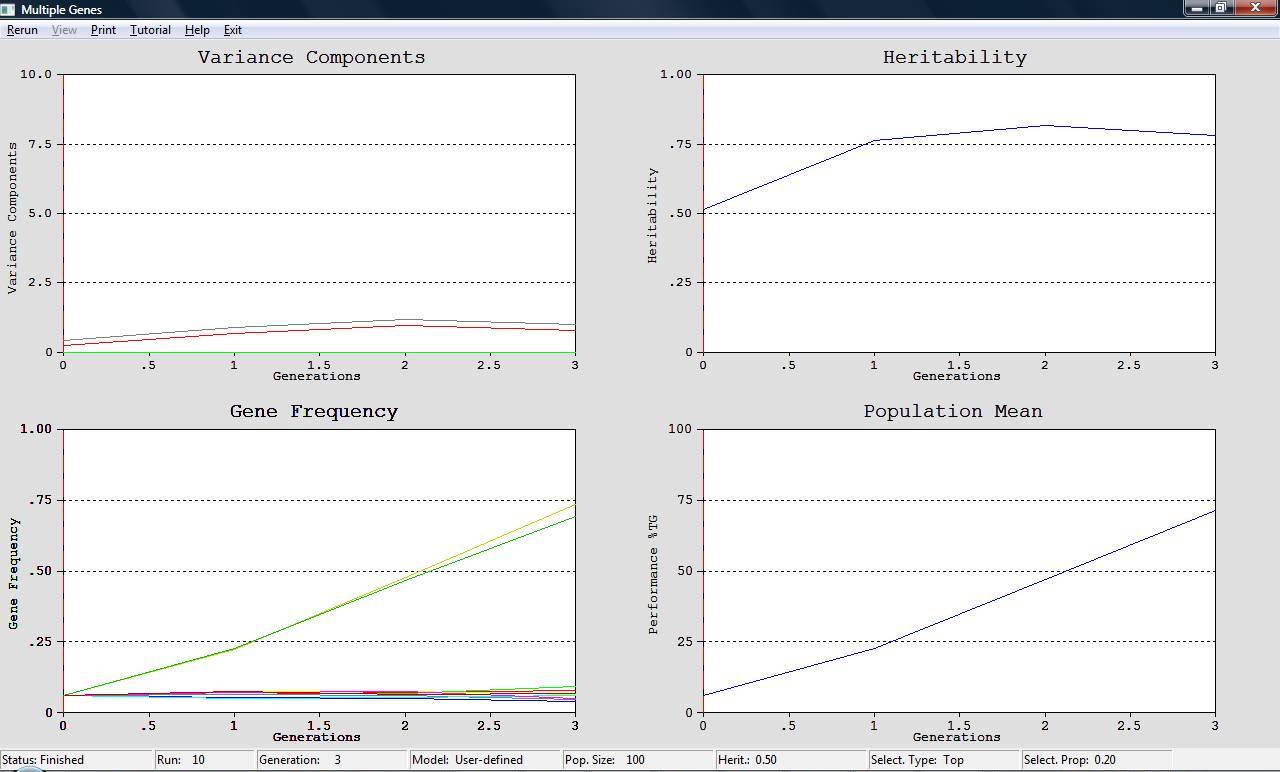
**

**Figure S2.** Response to selection simulated with QU-GENE model under the hypothesis of completely additive model (Podlich and Cooper 1998). Three generations of recurrent selection, two additive genes, population size of 100 plants, initial gene frequency of 0.06 (population mean as plant survival in the unselected population VLR1 at 375 g diclofop-methyl ha^-1^), selection proportion 0.2, heritability 50% and three generations of recurrent selection.


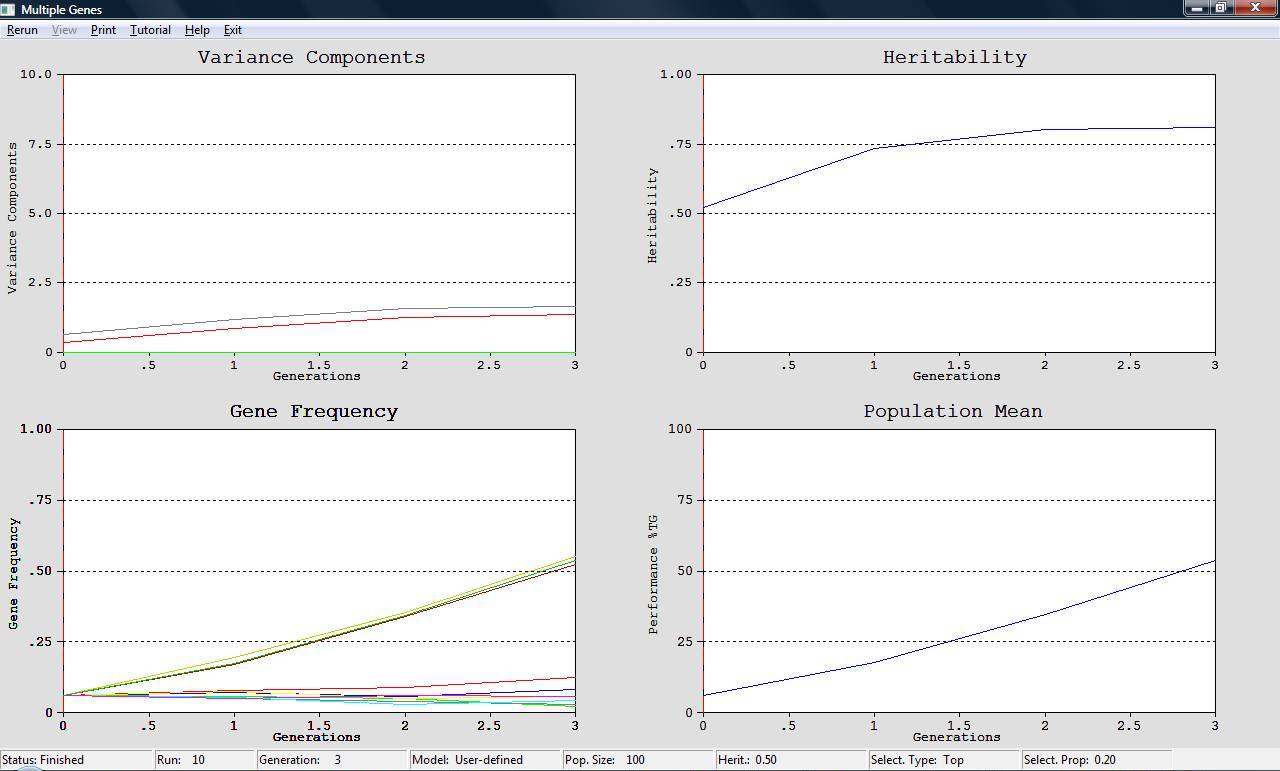


**Figure S3.** Response to selection simulated with QU-GENE model under the hypothesis of completely additive model (Podlich and Cooper 1998). Three generations of recurrent selection, three additive genes, population size of 100 plants, initial gene frequency of 0.06 (population mean as plant survival in the unselected population VLR1 at 375 g diclofop-methyl ha^-1^), selection proportion 0.2, heritability 50% and three generations of recurrent selection.


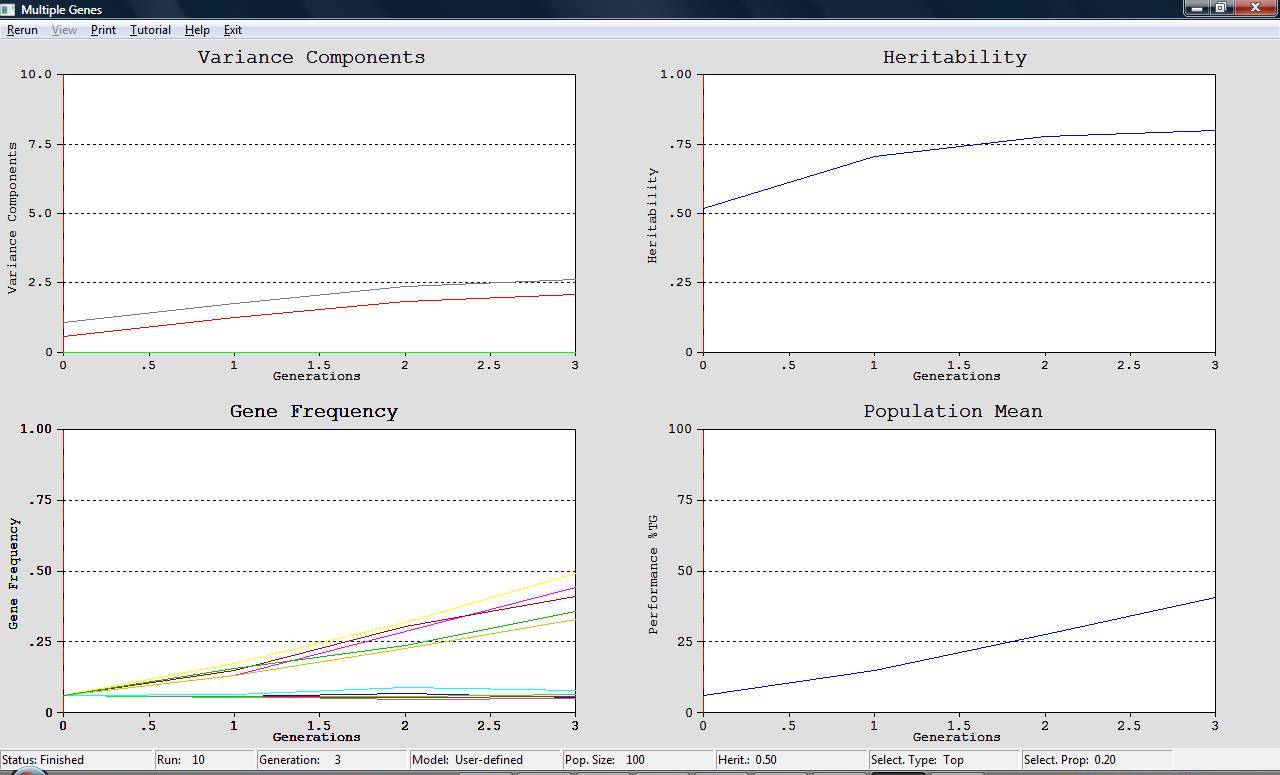


**Figure S4.** Response to selection simulated with QU-GENE model under the hypothesis of completely additive model (Podlich and Cooper 1998). Three generations of recurrent selection, five additive genes, population size of 100 plants, initial gene frequency of 0.06 (population mean as plant survival in the unselected population VLR1 at 375 g diclofop-methyl ha^-1^), selection proportion 0.2, heritability 50% and three generations of recurrent selection.


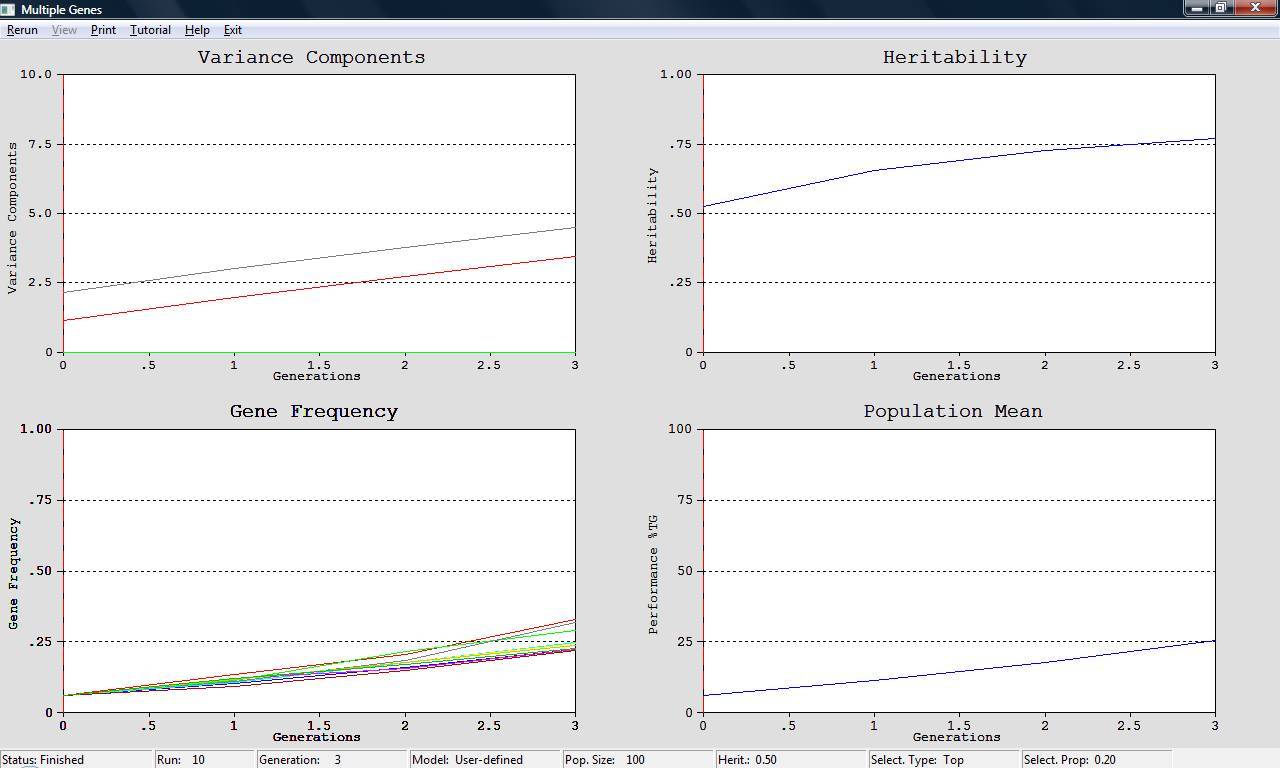


**Figure S5.** Response to selection simulated with QU-GENE model under the hypothesis of completely additive model (Podlich and Cooper 1998). Three generations of recurrent selection, ten additive genes, population size of 100 plants, initial gene frequency of 0.06 (population mean as plant survival in the unselected population VLR1 at 375 g diclofop-methyl ha^-1^), selection proportion 0.2, heritability 50% and three generations of recurrent selection.
