# Supplementary material for: Evolved polygenic herbicide resistance in Lolium rigidum by low-dose herbicide selection within standing genetic variation
Source: Evol Appl. 2012 Jul 12;6(2):231–42. doi: 10.1111/j.1752-4571.2012.00282.x (PMC3689349; doi:10.1111/j.1752-4571.2012.00282.x)
Supplement: Supplementary file 3 [file eva0006-0231-SD3.docx]

**Evolved polygenic herbicide resistance in *Lolium rigidum* by low-dose herbicide selection within standing genetic variation**

**Supplementary material**

**Materials and Methods**

Modeling simulations to explore the genetic basis of diclofop-methyl resistance evolution were conducted using a simplified version of the polygenetic platform QU-GENE to investigate the theoretical foundations and the characteristics of the genetic traits endowing diclofop-methyl resistance selected at sub-lethal doses of diclofop-methyl. A set of fixed genetic parameters were used to conduct the simulations as previously described in our earlier work: a) complete additive model with a default value of one, equally given to each allele, b) three generations of selection (displayed on the *x* axis of each graph) to simulate the recurrent selection study, c) ten simultaneous runs of the model (i.e. 10 lines displayed in the graphs), d) population size prior to selection of 100 plants and e) selection proportion equal to 0.20 (i.e. approximately the selection intensity applied under diclofop-methyl recurrent selection at). The graphs presented (below) display the components of variance, the heritability (narrow sense on individual basis), the gene frequency of the favorable alleles and the accumulated population mean. The initial gene frequency was set as 0.07, corresponding to the average plant survival in the unselected parent population (VLR1) obtained at 375 g diclofop-methyl ha^-1^ in that final herbicide bioassay. This diclofop-methyl rate was chosen because it is the recommended label rate used in field application of this herbicide. It clearly shows the effects of the recurrent selection on the evolution of diclofop-methyl resistance as progressive gene accumulation in the selected progenies as incremental increase in plant survival. Therefore, the validation of the potential genetic control is by comparison of different modelled scenarios (i.e. different number of genes enriched and heritability values) and the correspondence and similarity with empirical data (plant survival at a specific dose) obtained in the dose-response study with diclofop-methyl. The hypothesis of two, three five or ten additive genes were simulated and compared to establish the potential polygenic control of the resistance trait(s) selected.

**Results**

In the glasshouse experiment we compared all the diclofop-selected progenies with the unselected parent VLR1 to be able to assess the evolutionary dynamics of low-dose selected diclofop-methyl resistance under the same environment. By simple visual analysis and comparison of the graphs (Fig. S2-S3) it appears evident that two or three genes, associated with the evolved diclofop-methyl resistance, resulted in a simulated population mean (% performance) similar to the mean resistance frequency (% plant survival) obtained in the herbicide dose-response study of selected progenies at 375 g diclofop-methyl ha^-1^ (Fig. S1). By contrary the assumption of a greater number of resistance genes (i.e five or ten genes) (Fig.S4-S5) would have resulted in a much slower herbicide resistance evolutionary outcome and lower simulated population mean.
